# Pulmonary parenchymal Castleman tumor with fissural extension- a rare indication for pneumonectomy

**DOI:** 10.1186/1749-8090-10-S1-A5

**Published:** 2015-12-16

**Authors:** Krishnanand R Pai, Abilash Sathyanarayanan, C Ganesan, Vidhyalakshmi, MS Murugan, Prasanth A Biradhar, PR Murugesan

**Affiliations:** 1 Department of Cardiothoracic and Vascular Surgery, PSG Institute of Medical Sciences and Research, Coimbatore, India; 2 PSG Institute of Medical Sciences and Research, Coimbatore, India; 3 Department of Anaesthesiology, PSG Institute of Medical Sciences and Research, Coimbatore, India; 4 Department of Pathology, PSG Institute of Medical Sciences and Research, Coimbatore, India

## Background/Introduction

Castleman disease is a lymphoproliferative disorder of lymph tissue. Albeit benign there is a tendency to transform into lymphoma in some cases.

## Aims/Objectives

We present a rare case of Castleman tumour presenting as a solitary pulmonary tumor with extension into the oblique fissure on the left lung necessitating a pneumonectomy.

## Method

A 20 year fit and well lady presenting with constant inter-scapular back pain of 4 months, left hilar shadow on chest roentgenogram and CT scan revealing a centrally based left hilar mass originating from left upper lobe. There was no evidence of mediastinal lymphadenopathy, secondaries or enlargement of lymph nodes in the rest of the body.

## Results

Patient was taken up for surgery. Thoracotomy revealed a firm well encapsulated 4*4 cm, highly vascular, mass in close relation to hilar structures, crossing the oblique fissure into the lower lobe and with no invasion of hilar structures, or mediastinal lymphadenopathy. The mass was removed en bloc with a pneumonectomy. Histopathology confirmed a hyaline vascular type of Castleman tumor of lung.

## Discussion/Conclusion

Isolated pulmonary parenchymal Castleman disease is extremely rare and reported in less than 10 case reports worldwide. It commonly presents as a central mass and requires en bloc resection along with lobectomy/pneumonectomy. The potential for malignant transformation is justification for surgery albeit most cases are only diagnosed post resection. This is an important differential diagnosis for a well circumscribed, benign, centrally placed tumor of the lung and is a justifiable rare indication for pneumonectomy.

## Consent

Written informed consent was obtained from the patient for publication of this abstract and any accompanying images. A copy of the written consent is available for review by the Editor of this journal.

